# Interacting epidemics? Sleep curtailment, insulin resistance, and obesity

**DOI:** 10.1111/j.1749-6632.2012.06655.x

**Published:** 2012-07-24

**Authors:** Eliane A Lucassen, Kristina I Rother, Giovanni Cizza

**Affiliations:** 1 Immunogenetics Section, Clinical Center, National Institutes of HealthBethesda, Maryland; 2 Laboratory of Neurophysiology, Department of Molecular Cell Biology, Leiden University Medical CenterLeiden, the Netherlands; 3 Section on Pediatric Diabetes and Metabolism, National Institute of Diabetes and Digestive and Kidney Diseases, National Institutes of HealthBethesda, Maryland; 4 Section on Neuroendocrinology of Obesity, National Institute of Diabetes and Digestive and Kidney Diseases, National Institutes of HealthBethesda, Maryland

**Keywords:** sleep, obesity, insulin resistance, diabetes, appetite

## Abstract

In the last 50 years, the average self-reported sleep duration in the United States has decreased by 1.5–2 hours in parallel with an increasing prevalence of obesity and diabetes. Epidemiological studies and meta-analyses report a strong relationship between short or disturbed sleep, obesity, and abnormalities in glucose metabolism. This relationship is likely to be bidirectional and causal in nature, but many aspects remain to be elucidated. Sleep and the internal circadian clock influence a host of endocrine parameters. Sleep curtailment in humans alters multiple metabolic pathways, leading to more insulin resistance, possibly decreased energy expenditure, increased appetite, and immunological changes. On the other hand, psychological, endocrine, and anatomical abnormalities in individuals with obesity and/or diabetes can interfere with sleep duration and quality, thus creating a vicious cycle. In this review, we address mechanisms linking sleep with metabolism, highlight the need for studies conducted in real-life settings, and explore therapeutic interventions to improve sleep, with a potential beneficial effect on obesity and its comorbidities.

## Introduction

According to the National Health and Nutrition Examination Survey (NHANES) from 2009 to 2010, 33% of adult Americans were overweight (body mass index (BMI) of 25.0–29.9 kg/m^2^), and an even larger proportion (36%) were obese (BMI ≥ 30.0 kg/m^2^).^1^ The report contained good and bad news: for more than a decade, the age-adjusted prevalence of obesity had not changed in women overall, but there were marked ethnic, gender, and socioeconomic differences. Mexican American and Black women and men continued to show a significant rise in the prevalence of obesity. These figures are reflected in important obesity-related complications, such as hypertension, hyperlipidemia, and type 2 diabetes, all leading causes of cardiovascular disease.^2^ Type 2 diabetes affects 8.3% of the U.S. population (27% of U.S. residents aged 65 years and older), whereas 35% of U.S. adults have fasting glucose and glycosylated hemoglobin (HbA1c) levels in the prediabetic range.^3^ Thus, it is of great importance to characterize contributing etiological factors in order to effectively counteract the obesity and diabetes epidemics. The exact mechanisms explaining the relationship between short sleep, obesity, and diabetes remain unclear.

Accumulating evidence has pointed to an association between sleep curtailment and obesity,^4^,^5^ type 2 diabetes,^6^–^9^ and possibly mortality (see Ref. 10 for a review). In the last 50 years, self-reported sleep duration in the United States has decreased by 1.5–2 hours due to lifestyle changes in our society.^11^ Worldwide, secular trends in adult sleep duration may be more variable. A recent review article summarizing 12 studies from 15 countries from 1960 to 2000 did not find a consistent decrease in self-reported adult sleep duration: self-reported sleep duration had increased in seven countries, decreased in six, and results were inconsistent for the United States and Sweden.^12^ Imprecision in assessing sleep duration, as well as socioeconomic and demographic factors may play a role in this heterogeneity.

In this paper, we will first present the epidemiologic evidence for these relationships; we will then focus on sleep physiology and its role in metabolic functions. Then, we will critically evaluate the studies of sleep manipulation on glucose metabolism and energy expenditure (EE). We will highlight endocrine parameters that may influence these processes, including thyroid hormones, growth hormone (GH), cortisol, sympathovagal balance, and the immune system. We will also delineate the relationships between short sleep and some of the hormones that control appetite, including leptin, ghrelin, orexin, and neuropeptide Y (NPY). In addition, we will present evidence for the reverse effect: of obesity/diabetes on sleep. Finally, we will describe emerging evidence for a role of melatonin in the sleep–metabolism relationship and its therapeutic potential in metabolic disturbances.

## Epidemiologic evidence for an association among sleep duration, obesity, and diabetes

A meta-analysis of 45 cross-sectional studies, including 604,509 adults and 30,002 children, confirmed the relationship between short sleep (generally fewer than five hours per night in adults and fewer than 10 hours per night in children) and obesity, and quantified the associated risk (OR 1.55; 1.43–1.68; OR 1.89; 1.46–2.43, for adults and children, respectively).^13^ A similar OR of 1.58 (1.26–1.98) was found in children in another meta-analysis.^14^ Most of the included studies used actigraphy or self-reported sleep duration. A polysomnography study, not included in these meta-analyses, of 2,700 men above the age of 65 years revealed an inverse relationship between slow-wave sleep (SWS) duration and BMI or waist circumference.^15^ Another study reported that obese adults (BMI 41 ± 1 kg/m^2^) without sleep apnea slept 88 min fewer than lean subjects.^16^ The relationship between short sleep and BMI was also confirmed by actigraphy in 612 participants (35–50 years old).^17^

In principle, causality is best addressed in prospective randomized controlled studies. This ideal approach may not be practical in this case: studies of sleep extension or curtailment cannot be blinded, and participants may not behave according to the allocation group (i.e., control subjects changing sleep duration or, vice versa, intervention subjects not changing sleep duration). For these reasons, the existing prospective studies have all been observational in nature. Short sleepers experience greater weight increases over time according to most,^4^,^18^–^22^ but not all studies.^17^,^23^,^24^ However, average weight gain was modest and of relative clinical meaning. Short sleepers (fewer than five to six hours) gained 2 kg more in a 6-year study,^19^ and 0.4 kg more in another 16-year study.^18^ In addition, subjects had a 35% and 31% greater chance of gaining more than 5 kg in 6 years, and gaining more than 15 kg in 16 years, respectively.^18^,^19^ Thus, there is a large variability among studies in weight gain associated with short sleep for unknown reasons: the magnitude of this effect may be gender dependent or depend on other yet-to-be identified genetic and environmental factors. For example, short sleep may affect metabolism differently in natural “short sleepers” versus chronically sleep-deprived individuals. A large study including 35,247 subjects found that lean men, but not lean women, who slept fewer than five hours had increased odds of becoming overweight within one year (OR 1.91; 1.36–2.67).^25^ In contrast, in a cohort of 3576 elderly subjects, women, but not men, who slept fewer than five hours, had a higher risk of gaining 5 kg in two years (OR 3.41; 1.31–8.69).^20^ Interestingly, in this study, BMI was also increased in women sleeping more than eight hours. A similar *U*-shaped association has been reported in other studies.^19^,^25^ Collectively, these studies indicate that self-reported short or long sleep is associated with increased BMI. The amount of weight gain over time, however, varied greatly, ranging from less than 1 kg to several kg, and age may be one of the parameters modulating this association.

Many mechanisms may be responsible for the frequently observed insulin resistance in obese individuals, including increased release of free fatty acids, leptin, and TNF-α from adipose tissue.^26^ We suggest that short sleep (fewer than five to six hours) may now qualify as an additional clinical factor in the development of insulin resistance and diabetes, as shown in several cross-sectional studies.^27^–^29^ A recent study of 174,542 middle-aged subjects showed that short sleepers had an OR of 1.46 (1.31–1.63) for developing diabetes after 3–10 years of follow-up.^30^ A meta-analysis of 10 prospective studies reported a pooled OR of 1.28 (1.03–1.60) for diabetes in short sleepers.^31^ Interestingly, this correlation was only present in men (OR 2.07; 1.16–3.72). Both long sleep (OR 1.48; 1.13–1.96) and decreased sleep efficiency also resulted in greater chances of developing diabetes.^31^ Difficulty in falling asleep resulted in a greater incidence of diabetes after 4.2–14.8 years of follow up.^32^–^34^ In smaller studies, the association between sleep duration and diabetes is more variable.^7^–^9^,^35^ In addition, this variability may be real and differ on the basis of ethnic group and socio-economic status: a study found increased risk of developing diabetes only in Whites and Hispanics who slept fewer than seven hours.^36^ Besides sleep-duration, poor sleep quality relates to insulin resistance. Frequency of nocturnal awakenings, as measured by actigraphy, correlated positively with HbA1c levels in patients with type 2 diabetes.^37^ Furthermore, sleep disordered breathing was related to worse blood glucose control.^38^ Taken together, there is solid epidemiologic evidence for an interconnection between sleep duration (too short or too long), insulin resistance, and obesity.

## Physiological changes of metabolism during normal sleep

**Figure 1:**
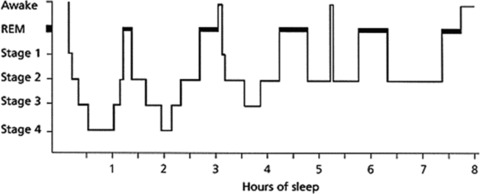
Representative sleep architecture during eight hours of uninterrupted sleep (adapted from Ref. 39). Sleep can be divided into rapid eye movement (REM) and nonrapid eye movement (NREM) sleep. On average, adults spend 75–80% in NREM sleep, which can be further subdivided into stages 1 to 4. Stages 3 and 4 are often referred to as slow-wave sleep (SWS) because of the appearance of well-defined waves of 0.5–4.0 Hz. In REM sleep, a phasic and a tonic phase can be distinguished. Skeletal muscles are atonic or hypotonic during REM sleep (except for the diaphragm, extraocular, and sphincter muscles), but bursts of muscle activity can occur during the phasic phase. Furthermore, dreams mostly take place during REM sleep and are more complex during this stage. Eye movement is only present during the phasic phase. Throughout a normal night of sleep, there are three to five sleep cycles, from NREM (stage 1 to 4) to REM, each cycle taking 90–120 minutes. Humans display less SWS and more REM sleep toward the end of the night.

Sleep architecture, the structure and pattern of sleep, is best described by a combined approach that uses electroencephalographic, electromyographic, and other criteria^39^ (Fig. 1). Many metabolic parameters display diurnal rhythms, which are the resultant of both homeostatic and circadian mechanisms. From a homeostatic point of view, sleep pressure increases with sustained wakefulness. Circadian rhythms are endogenously generated in the suprachiasmatic nucleus of the hypothalamus by feedback loops of transcripts of a group of “core clock” genes, including the CLOCK gene.^40^,^41^ Time-keeping signals are passed on from the suprachiasmatic nucleus to oscillators in peripheral tissues also containing core clock genes. Endogenous rhythms, in turn, are synchronized to the outside world by various environmental clues, mostly by light via the retina.

**Figure 2:**
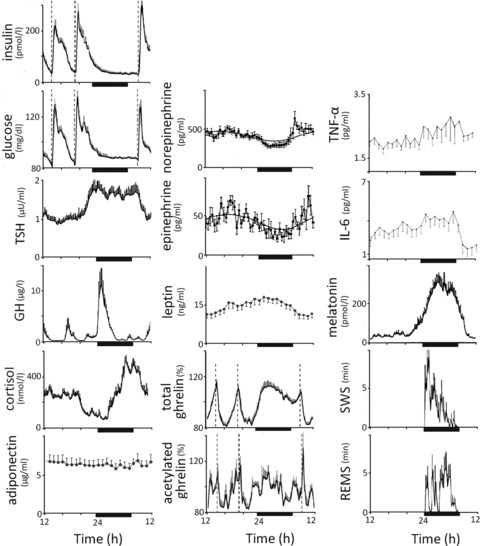
Mean levels of metabolic parameters and sleep stages in lean young men (*n* = 8 for TSH, cortisol, GH, melatonin, SWS, and REM sleep;^48^*n* = 14 for insulin, glucose, total and acetylated ghrelin;^76^*n* = 8 for catecholamines^73^); in 23 lean women for leptin and adiponectin;^69^ and in 25 individuals (13 females, 12 males) for IL-6 and TNF-α.^83^ Ghrelin levels are indicated as a percent of mean 24-h values (1027 pg/mL for total ghrelin; 80 pg/mL for acetylated ghrelin); leptin levels are shown as percent change from levels at 08:00. Dark bars below each plot indicate bedtimes. Sleep was monitored polysomnographically during all measurements. Striped bars indicate meal times. Modified, with permission Refs. 48, 69, 73, 76, and 83

During sleep, energy requirements are lower as metabolic needs for processes such as breathing, gut motility, heart rate and muscular activity, are decreased.^42^ Indeed, resting EE, as determined by indirect calorimetry, was lower in healthy volunteers during sleep compared to wakefulness.^43^ During sleep glucose levels remain stable or fall only minimally in spite of fasting, mostly because of decreased energy needs.^44^ In contrast, if subjects are kept awake in bed while fasting for a comparable number of hours, glucose levels do fall, approximately 15 mg/dl.^45^ During the first part of the night, when most slow wave sleep (SWS) occurs, glucose production is diminished^44^ (Fig. 2). Brain glucose metabolism, as measured by PET, decreases by 30–40%.^46^,^47^ During the second half of the night, glucose consumption increases due to increased REM sleep requiring more energy.^46^,^47^

EE and glucose metabolism are modulated by a number of hormones. Thyroid hormones are important determinants of EE; cortisol and GH are powerful modulators of circadian rhythms of glucose metabolism. The sympathovagal system and adiponectin influence both EE and glucose metabolism. Thyroid stimulating hormone (TSH) levels rise at night, mostly due to increased hypothalamic secretion of thyroid releasing hormone (TRH) (Fig. 2).^48^

GH secretion is under dual hypothalamic control: somatostatin is inhibitory and growth hormone releasing hormone (GHRH) is stimulatory. Somatostatin and GHRH are secreted in alternation, thus generating the typical pulsatile pattern of GH secretion. GH typically peaks at sleep onset during SWS (Fig. 2).^49^ GH levels were lower in SWS-deprived individuals^50^ and higher in individuals with pharmacologically induced SWS.^51^ In addition, REM sleep deprivation did not affect plasma GH levels.^52^ Intravenous GH administration decreases SWS, suggesting a modulatory role for GH on SWS.^53^,^54^ The GH peak at sleep onset has insulin lowering effects.^55^ GHRH injection increased glucose levels at awakening by almost 50%^56^ and GH administration rapidly decreased muscular glucose uptake.^55^,^57^

Cortisol concentrations reach their zenith in the morning, experience a gradual fall during the day, which is briefly interrupted by meals, and have their nadir around 3 am (Fig. 2).^48^ Cortisol impairs insulin sensitivity with a latency of four to six hours.^56^,^58^,^59^ Plasma insulin levels also display a modest diurnal variation with a 10% excursion, a nadir between midnight and 6 am and a peak between noon and 6 pm.^60^

Adiponectin is a hormone secreted by the adipocytes in an inverse fashion compared to fat mass.^61^ Its role in energy homeostasis is unclear: increases, decreases, or no changes in energy balance are derived from peripheral adiponectin administration in rodents.^62^ Adiponectin knockout mice develop insulin resistance and increased TNF-α levels when fed a high fat diet. In humans, plasma adiponectin levels correlated negatively with insulin resistance, and higher levels correlated with future weight gain in 1,063 women.^63^,^64^ Individuals with type 2 diabetes typically have low adiponectin levels, even when adjusted for body weight.^65^ In contrast, patients with type 1 diabetes have higher than expected adiponectin levels: an observation that is poorly understood.^66^ Sleep duration was inversely related to serum adiponectin in 109 lean Japanese males.^67^ Adiponectin has anti-inflammatory properties: *in vitro* exposure to adiponectin decreases macrophage activation and TNF-α production. Finally, adiponectin displays some diurnal variability, with lower levels at night (Fig. 2).^68^,^69^

During sleep, vagal tone increases.^70^ Sympathovagal balance is lowest during REM sleep, when the locus coeruleus, the main brain source of catecholamine biosynthesis, is silent.^71^ Plasma norepinephrine (NE) and epinephrine (EPI) are produced by the sympathetic system: EPI is secreted exclusively from the adrenal medulla, whereas NE can also be released from postganglionic sympathetic nerves.^72^ In a small study of eight lean males, plasma catecholamines were consistently lower during the night with no differences among sleep stages (Fig. 2).^73^ Heart rate and mean blood pressure were lower during NREM sleep, compared to during awakenings and REM sleep.^70^

Several hormones involved in appetite regulation display circadian rhythms. Leptin is secreted by the white adipose tissue in a highly pulsatile fashion.^74^ Leptin reflects fat stores: its levels increase after meals and during the night and are associated with decreased appetite (Fig. 2).^74^ Ghrelin, produced in the stomach, stimulates appetite and circulates mainly in a nonacetylated form.^75^ Acetylated ghrelin, the active form, is necessary for most of its endocrine actions, including GH release, appetite stimulation, and gastric emptying. Total and acetylated ghrelin levels rise with fasting and during sleep (Fig. 2).^76^ Leptin and ghrelin exert their effects in the arcuate nucleus of the hypothalamus, through anorexigenic proopiomelanocortin/cocaine- and amphetamine-regulated transcript (POMC/CART) and orexigenic neuropeptide Y/agouti-related protein (NPY/AgRP) neurons. Leptin activates POMC/CART neurons, which induces hunger and inhibits NPY/AgRP neurons,^77^,^78^ which induces satiety. Ghrelin has the opposite effect on the arcuate nucleus, stimulating appetite, and prolonging postprandial glucose responses, while stimulating GH release.^79^ Insulin, while peripherally lowering blood glucose and stimulating appetite, inhibits appetite centrally in a leptin-like manner.^78^

Orexin A and B, two neuropeptides released by lateral hypothalamic neurons, stimulate appetite. Orexin neurons are active during waking and quiescent during sleep; consistently, orexin levels in the CSF are maximal at the end of the waking period.^80^,^81^ The orexin system is activated by ghrelin and inhibited by leptin and glucose. Projections from orexin neurons in the lateral hypothalamus activate NPY neurons in the arcuate nucleus.^80^ Melatonin is a sleep-promoting hormone that is produced by the pineal gland. Melatonin is exclusively secreted during the dark, as light inhibits its production (Fig. 2).

Interleukin 6 (IL-6), interleukin 1β (IL-1β), TNF-α, and C-reactive protein (CRP) are pro-inflammatory factors. IL-6 displays a circadian rhythm, with a first peak around 2 am and a second peak around 5 am (Fig. 2).^82^,^83^ IL-1β decreases during the night and reaches its nadir at 8 am in the morning.^82^ TNF-α also displays a circadian rhythm, peaking close to the awakening (6 am) and reaching a nadir around 3 pm (Fig. 2). IL-6 and TNF-α stimulate secretion of cortisol and, in turn, cortisol inhibits their secretion.^84^ Reports on diurnal variability of CRP are few and inconsistent: some report no diurnal variance,^85^ whereas others find increased morning levels.^86^ In 10 healthy, lean men, white blood cell counts peaked around 11 pm, decreased throughout the night and reached a nadir at 8 am.^82^

The relationships here described between hormone secretion and sleep stages are necessarily descriptive. In theory, as new pharmacological tools aimed at manipulating hormonal status (i.e., selective hormone agonists/antagonists) and/or sleep become available for investigation, we may be able to better understand the numerous causal links interconnecting these phenomena. In summary, many metabolic parameters involved in EE regulation, glucose metabolism, and appetite control display diurnal rhythms, reflecting the different metabolic needs during the sleep and wake states. In the next section, we will report the effect of sleep deprivation on these metabolic parameters.

## Effects of sleep restriction on metabolic parameters

**Figure 3:**
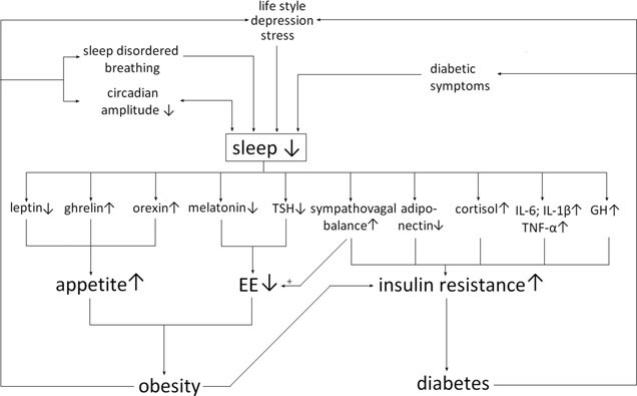
A simplified schematic representation of putative pathways of sleep curtailment leading to obesity and diabetes, via endocrine mechanisms that stimulate appetite, decrease energy expenditure, and increase insulin resistance. The direction of change (an increase or decrease) of each mechanism due to sleep loss is displayed; increased sympathovagal balance could stimulate EE, depicted by +, while a decreased EE would contribute to obesity. For readability, the relationships between decreases in leptin levels associated with IL-6 and TNF-α production are not displayed. Cortisol and proinflammatory cytokines display a positive bidirectional relationship. Increased insulin levels stimulate orexin secretion and orexic arcuate neurons, while decreasing the activity of anorexic arcuate neurons, leading to a further increase in appetite. Adiponectin influences energy homeostasis, possibly via modulating appetite through arcuate neurons and modulating EE.

The relationship between sleep and metabolism has been extensively studied in human subjects under varying conditions, such as total sleep deprivation,^87^–^100^ partial sleep deprivation,^101^–^122^ and sleep fragmentation^123^–^125^ (Table 1). Partial sleep deprivation affects SWS less than other sleep stages. Most of the experiments on the effects of acute sleep deprivation were conducted in healthy, lean volunteers. Food intake and physical activity were often, but not always, strictly controlled (Table 1). It is important to note that experimentally induced sleep alterations may not be representative of changes occurring in clinical condition of chronic sleep deprivation. In addition, important differences exist between acute and chronic sleep deprivation. We will describe effects of sleep curtailment on important metabolic parameters that may contribute to insulin resistance and obesity, including effects on appetite and EE (Fig. 3).

**Table 1:** Results of 37 human experimental studies assessing the influence of sleep curtailment on metabolism

Study design Energy intake Behavioral activity	Gender/sample size Age ± SEM (years) Mean weight ± SEM Prestudy conditions	Experimental sleep protocol	Techniques used for blood sampling (time, frequency)	Main findings (sleep deprived vs. longer sleep)
Benedict *et al.*^100^ Randomized cross-over study Controlled, postintervention *ad libitum* Bed rest	Men (*n* = 14) 22.6 ± 0.8 23.9 ± 0.5 kg/m^2^ 6 weeks regular bedtimes^*c*^	1: 1 night of 8-h bedtime^*e*^ 2: 1 night of TSD Conditions ≥ 4 weeks apart	Indirect calorimetry pre- and postbreakfast; morning VAS hunger ratings 24 h, every 1.5–3 h	↑ postprandial glucose (8%) ↓ RMR (5%) and postprandial (20%) metabolic rate ↑ cortisol (7%)*^a^*↑ NE (12%)*^a^*∼ food intake; ↑ hunger (60%)*^a^*↑ morning ghrelin (11%) ∼ leptin
Born *et al.*^82^ Randomized cross-over study Standardized meals Synchronous inpatient activities	Men (*n* = 10) 24.7 (21–29) NA NA	1: 1 night of 8-h bedtimes^*e*^ then 1 night of *ad libitum* sleep^*e*^ 2: 24 h TSD^*e*^ Conditions ≥ 10 days apart	LPS-stimulated whole blood and ELISA (TNF-α; IL-1β; IL-6) 51 h, every 3 h	∼ IL-6 ∼ IL-1β; ↑ nocturnal whole blood ∼ TNF-α↑ white blood cells (8%)^1^
Brondel *et al.*^113^ Randomized cross-over study *Ad libitum* Free physical activity^*d*^	Men (*n* = 12) 22 ± 3 22.3 ± 1.83 kg/m^2^ 2 days sleep/diet diaries^*c*^	1: 1 night of 8-h bedtime^*c*^ and 1 night *ad libitum* sleep at home^*c,d*^ 2: 1 night of 4-h bedtime^*e*^ and 1 night of free bedtimes at home^*c,d*^ Conditions ≥ 5 days apart	Actigraphy; 12 daytime VAS hunger ratings	∼ EE; ↑ physical activity (13%) ↑ hunger; ↑ food intake (22%)
Buxton *et al.*^114^ Consecutive phases study Controlled isocaloric (finishing meal required) Controlled sedentary living	Men (*n* = 20) 26.8 ± 5.2 23.3 ± 3.1 kg/m^2^≥ 5 days of 10-h bedtimes^*c,d*^	1: 3 nights of 10-h bedtimes^*d*^ 2: 7 nights of 5-h bedtimes^*d*^	ivGTT penultimate day; clamp last day; salivary sampling 15:00–21:00 last 2 days, every hour, 24-h urine sampling last 2 days; indirect calorimetry	↑ insulin resistance (20% by ivGTT; 11% by clamp) ∼ fasting RMR ↑ salivary cortisol (51%) ↑ urinary NE (18%) and EPI (22%)^1^
Clore *et al.*^44^ Comparative study Similar evening meals Bed rest	5 females, 12 males (*n* = 17) 25 ± 1 24.1 ± 0.6 kg/m^2^ NA	1. Group 1 (*n* = 11): 1 night of 8-h, 30 min bedtime 2. Group 2 (*n* = 6): 1 night of TSD	Continuous [^3–^3H] glucose infusion	↓ nocturnal fall in glucose use ↓ nocturnal fall in glucose production
Donga *et al.*^115^ Randomized cross-over study NA	3 females, 4 males (*n* = 9) 44 ± 7 23.5 ± 0.9 kg/m^2^	1: 3 nights of 8-h, 30-min bedtimes^*d*^	Hyperinsulinemic euglycemic clamp	↑ insulin resistance (21%)
NA	Type 1 diabetes, HbA1c ≤ 8.5%, 1 week of regular bedtimes^*c,d*^	2: 3 nights of 4-h bedtimes^*e*^ Conditions ≥ 3 weeks apart		
Dzaja *et al.*^96^ Randomized cross-over study 1,800 kcal/24 h Bed rest	Men (*n* = 10) 28 ± 3.1 24.0 ± 2.9 kg/m^2^ 1 week of regular bedtimes^*c*^	1: 1 night of 8-h bedtime^*e*^ 2: 1 night of TSD Conditions ≥ 2 weeks apart	24 h, every h	↓ GH peak (71%)*^b^*∼ mean cortisol levels ↓ nocturnal total ghrelin increase (39%)*^b^*
Frey *et al.*^96^ Consecutive phases study Hourly controlled intake Bed rest	9 females, 10 males (*n* = 19) 28.1 ± 8.6 18.5–24.5 kg/m^2^ 3 weeks of regular bedtimes^*c,d*^	1: 3 nights of 8-h bedtimes^*e*^ 2: 1 night of TSD	Saliva 36 h, every h; hsELISA (IL-6; IL-1β; CRP) 40 h, every 30 min	∼ saliva cortisol; ↓ at 13:00 (53%)*^b^* and 20:00 (17%)*^b^*↓ daytime IL-6 ↑ IL-1β↓ morning hsCRP
Gonzáles-Ortiz *et al.*^90^ Consecutive phases study Controlled isocaloric Free inpatient movement	7 females, 7 males (*n* = 14) 21.0 ± 2.1 22.1 ± 1.3 kg/m^2^ 3 days diet diary	1: baseline “normal sleep” 2: 1 night of TSD	Insulin suppression test with octreotride Once, morning	↑ insulin resistance (18%)*^a^*∼ morning cortisol
Haack *et al.*^108^ Comparative study Controlled isocaloric Free inpatient movement	6 females, 12 males (*n* = 18) 27.3 ± 5.8 23.1 ± 3.3 <30 kg/m^2^≥ 10 days regular bedtimes^*c*^	(*n* = 8) 1: 10 nights of 8-h bedtimes^*d*^ (*n* = 10) 2: 10 nights of 4-h bedtimes^*d*^	24-h urine 24-h blood, every 4 h (high sensitivity ELISA IL-6 and hsCRP)	∼ food intake ↑ IL-6 (62%)*^a^*∼ TNF-receptor 1 ↑ hsCRP (102%; *P* = 0.11)^1^
Hursel *et al.*^125^ Randomized cross-over study Controlled isocaloric Sedentary activity	Men (*n* = 15) 23.7 ± 3.5 24.1 ± 1.9 kg/m^2^ 2 days controlled diet	1. 2 nights of 8-h bedtimes^*e*^ 2: 2 nights of sleep fragmentation hourly 2-min–long alarms^*e*^ Conditions ≥ 2 weeks apart	Indirect calorimetry	∼ total EE ↑ physical activity (8%)*^a^*↑ RQ (3%)*^a^*
Irwin *et al.*^107^ Consecutive phases study NA No exercise	13 females, 17 males (*n* = 30) 37.6 ±9.8 <30 kg/m^2^ 2 weeks regular bedtimes^*c*^	1: 3 nights of 8-h bedtimes^*e*^ 2: 1 night of 4-h bedtime^*e*^	Flow cytometry; real time PCR (*n* = 10); high-density oligonucleotide array (*n* = 5) Daytime (08:00–23:00), every 3 to 4 h	↑ IL-6 monocyte expression (344%)*^a^*; ↑ IL-6 mRNA (250%)*^a^*↑ TNF-α monocyte expression (87%)*^a^*; ↑ TNF-α mRNA (100%)*^a^*
Jung *et al.*^43^ Consecutive phases study Controlled isocaloric Sedentary	2 females, 5 males (*n* = 7) 22.4 ± 4.8 22.9 ± 2.4 kg/m^2^ 1 week 8-h sleep^*c,d*^;3 days diet	1: 2 nights of 8-h bedtimes^*e*^ 2: 40 h of TSD	Indirect calorimetry	↑ total EE (7%); ↑ nocturnal EE (32%); ∼ daytime EE ∼ RQ
Kuhn *et al.*^87^ Consecutive phases study Balanced diet NA	Human subjects (*n* = 28) 20–30 NA	1: 4–5 days “control period” 2: 72–126 h of TSD 3: 3 days “control period”	OGTT Once daily	↓ glucose tolerance after 3–4 days ↑ 17-OH corticosteroids on Day 2 and 3, then return to baseline ↑ urinary catecholamines
Mullington *et al.*^94^ Consecutive phases study Controlled isocaloric (finishing not required) Sedentary	Men (*n* = 10) 27.2 26.1 ± 1.9 kg/m^2^ 1 week of regular bedtimes^*c,d*^	1. 3 nights 8-h bedtimes^*e*^ 2. 3 nights of TSD	120 h, every 90 min	∼ leptin mesor, ↓ leptin circadian amplitude
Nedeltcheva *et al.*^109^ Kessler *et al.*^116^ Randomized cross-over study *Ad libitum* Sedentary	5 females, 6 males (*n* = 11) 39 ± 5 26.5 ± 1.5 kg/m^2^ NA	1: 14 nights of 8.5-h bedtimes^*e*^ 2: 14 nights of 5.5-h bedtimes^*e*^ Conditions ≥ 3 months apart	OGTT; ivGTT 24 h, every 15–30 min	↓ insulin sensitivity (18%)*^a^*↓ TSH (7%; especially in females)^*a*^↓ free T4 (8%)*^a^*∼ GH; ↓ during first 4-h of bedtime period in males (31%)*^a^*∼ cortisol; ↓ and 44 min later acrophase (10%)*^a^*; 71 min later nadir ↑ EPI (21%)*^a^*∼ mean NE; ↑ nocturnal (24%)*^a^*
Nedeltcheva *et al.*^118^ Randomized cross-over study Controlled restricted (90% RMR) Sedentary	3 females, 7 males (*n* = 10) 41 ± 5 27.4 ± 2 kg/m^2^ NA	1: 14 nights of 8.5-h bedtimes^*e*^ 2: 14 nights of 5.5-h bedtimes^*e*^ Conditions ≥ 3 months apart	Indirect calorimetry; VAS hunger rating scale before meal/at 22:30, 24 h, every hour	∼ total EE; ↓ RMR (8%)*^a^*; ↑ fasting and postprandial RQ (4%)*^a^*∼ thyroid hormones ∼ GH ∼ cortisol ↓ EPI (12%)*^a^*; ∼NE ∼ food intake; ↑ hunger ∼ total weight loss (3 kg), ↓ fat weight loss (55%), ↑ fat-free weight loss (60%) ↑ mean acylated ghrelin (7%)*^a^*∼ leptin
Omisade *et al.*^117^ Consecutive phases study Controlled Free inpatient movement	Women (*n* = 15) 21.6 ± 2.2 24.5 ± 8 kg/m^2^ 1 week regular bedtimes and diet^*c,d*^	1: 1 night of 10-h bedtime^*d*^ 2: 1 night of 3-h bedtime^*d*^	VAS hunger ratings; salivary cortisol daytime, every 1–2 h; salivary leptin morning and afternoon	↓ median morning cortisol (19%)*^a^*; slower decline; ↑ afternoon/evening (44%)*^a^*∼ hunger ↑ morning leptin (8%)*^a^*
Parker *et al.*^88^ Consecutive phases study Controlled Sedentary	Men (*n* = 4) 21–30 NA NA	1: 1 night of 8-h bedtime^*e*^ 2: 2 nights of TSD	72 h, every 30 min	↑ TSH; ↑ amplitude, longer peak, later nadir/acrophase
Pejovic *et al.*^99^ Consecutive phases study *Ad libitum* Ambulatory	11 females, 10 males (*n* = 21) 24.1 ± 3.1 24.1 ± 2.6 kg/m^2^ 2 weeks regular bedtimes^*d*^	1: 4 nights of 8-h bedtimes^*e*^ 2: 1 night of TSD, 50% had 2-h afternoon nap	VAS hunger ratings 24 h, every 30 min	∼ cortisol ∼ adiponectin ∼ hunger ↑ leptin (14%)*^a^*
Schmid *et al.*^97^ Randomized cross-over study None Bed rest	Men (*n* = 10) 25.3 ± 1.4 23.8 ± 0.5 kg/m^2^ Light dinner before arrival in the lab (21:00)	1: 1 night of 8-h bedtime^*e*^ 2: 1 night of TSD Conditions ≥ 2 weeks apart	Hypoglycemic clamp, Two morning samples	↓ glucagon levels (16%)*^a^*; ↑ response to hypoglycemia ↓ cortisol levels (16%)*^a^*; ∼ ACTH ∼ catecholamines ↑ hunger
Schmid *et al.*^98^ Randomized cross-over study None Bed rest	Men (*n* = 9) 24.2 ± 1.0 23.8 ± 0.6 kg/m^2^ NA	1: 1 night of 7-h, 30-min bedtime^*e*^ 2: 1 night of 5-h bedtime^*e*^ 3: 1 night of TSD Conditions ≥ 2 weeks apart	Hunger graded 0–9, Two morning samples	↑ hunger (129%)*^a^*∼ leptin ↑ total ghrelin (22%)
Schmid *et al.*^110^ Schmid *et al.*^119^ Randomized cross-over study *Ad libitum* Free outpatient movement	Men (*n* = 15) 27.1 ± 1.3 22.9 ± 0.3 kg/m^2^ 4 weeks regular bedtimes^*c*^	1: 2 nights of 8-h bedtimes^*e*^ 2: 2 nights of 4-h bedtimes^*e*^ Conditions ≥ 6 weeks apart	Actigraphy, buffet on day 2, ELISA (IL-6) 08:00–23:00 (every h)	↑ insulin (40%) and glucose (11%) peak response to breakfast ↓ physical activity (13%)*^a^*∼ cortisol; ∼ ACTH ∼ total energy intake; ↑ fat intake (23%)*^a^*∼ hunger; ∼ intake ∼ leptin; ∼ ghrelin ∼ IL-6
Schmid *et al.*^111^ Randomized cross-over study None Bed rest	Men (*n* = 10) 25.3 ± 1.4 23.8 ± 0.5 kg/m^2^ 2 weeks regular bedtimes^*c*^	1: 1 night of 7-h bedtime^*e*^ 2: 1 night of 4.5-h bedtime^*e*^ Conditions ≥ 2 weeks apart	Hypoglycemic clamp 2 morning samples	∼ insulin resistance ↓ glucagon (8%)*^b^*∼ GH ↓ ACTH (44%)*^a^*; ↓ cortisol (44%)*^a^*∼ EPI; ∼ NE
Shearer *et al.*^93^ Comparative study Controlled isocaloric No exercise	Men (*n* = 42) 28.7 (21–47) NA 2 weeks regular bedtimes^*d*^	(*n* = 21) 1: 3 nights of 8-h bedtimes, then 4 nights of 2-h bedtimes (*n* = 21) 2: 3 nights of 8-h bedtimes, then 4 nights of TSD	5 days, every 6 h (ELISA)	No changes in condition 1; in condition 2: ↑ IL-6 ∼ TNF-α↑ sTNF-α receptor I ∼ sTNF-α receptor II
Simpson *et al.*^120^ Consecutive phases study *Ad libitum* Free inpatient movement	67 females, 69 males (*n* = 136) 30.4 (22–45) 24.7, 17.7–32.6 kg/m^2^ 1 week regular bedtimes^*c,d*^	1: 2 nights of 10-h bedtimes 2: 5 nights of 4-h bedtimes	Once, morning (10:30–12:00)	↑ leptin (33%)*^a^*
Simpson *et al.*^121^ Consecutive phases study *Ad libitum* Free inpatient movement	33 females, 41 males (*n* = 74) 29.9, 22–45 24.6, 17.7–33.1 kg/m^2^ 1 week regular bedtimes^*c,d*^	1: 2 nights of 10-h bedtimes^*e*^ 2: 5 nights of 4-h bedtimes^*e*^	Once, morning (10:30–12:00)	∼ adiponectin in men; ↑ in African American women; ↓ in Caucasian women
Spiegel *et al.*^101^ Spiegel *et al.*^102^ Spiegel *et al.*^103^ Spiegel *et al.*^104^ Consecutive phases study Controlled Free outpatient movement	Men (*n* = 11) 22 ± 1 23.4 ± 0.5 kg/m^2^≥ 1 week of 8-h bedtimes, diet^*d,e*^	1: 6 nights of 4-h bedtimes^*e*^ 2: 6 nights of 12-h bedtimes^*e*^	ivGTT; 24-h heart rate variability; salivary sampling 15:00-bedtime, every 30 min; blood sampling 24 h, every 30 min	↓ glucose clearance (30%) ↓ TSH (26%), ↑fT4 (7%)*^a^*∼ GH, different profile ↑ afternoon saliva/plasma cortisol (25%)*^a^*↑ sympathovagal balance (17%)*^a^*↓ leptin (19%); ↓ and 2.5 h earlier acrophase (26%); ↓ amplitude (20%) ↓ melatonin; later onset; ↓ acrophase
Spiegel *et al.*^105^ Randomized cross-over study Controlled Free outpatient movement	Men (*n* = 12) 22 ± 2 23.6 ± 2.0 ≥ 1 week of 8-h bedtimes	1: 2 nights of 10 h bedtimes^*e*^ 2: 2 nights of 4-h bedtimes^*e*^ Conditions ≥ 6 weeks apart	VAS hunger ratings 08:00–21:00, every 20 min	↑ hunger (23%; 33–45% for sweets/salty foods) ↓ leptin (18%) ↑ ghrelin (28%)
Stamatakis *et al.*^123^ Consecutive phases study *Ad libitum* NA	2 females, 9 males (*n* = 11) 23.2 (18–29) 24.3 ± 0.9 kg/m^2^ 3 days diet and >7-h bedtimes^*a,b*^	1: 1 night of unfragmented sleep^*e*^ 2: 2 nights of fragmented sleep (∼ 31.4 auditory/mechanical stimuli per hour)^*e*^	ivGTT; heart rate variability; enzyme-linked immunosorbent assay techniques (IL-6; hsCRP) 2 morning samples (08:00 and 16:00)	↑ insulin resistance (25%) ↑ morning cortisol (13%) ∼ adiponectin ↑ sympathetic tone (17%) ∼ leptin ∼ Il-6; ∼hsCRP
St-Onge *et al.*^122^ Randomized cross-over study Controlled isocaloric, self-selected onday 5 Free inpatient movement	15 females; 15 males (*n* = 30) 33.9 ± 4.3; 36.6 ± 5.6 23.0 ± 1.1; 24.1 ± 1.1 kg/m^2^ 2 weeks regular bedtimes^*c,d*^	1: 5 nights of 9-h bedtimes^*e*^ 2: 5 nights of 4-h bedtimes^*e*^ Conditions ≥ 4 weeks apart	Double-labeled water (EE); indirect calorimetry (RMR); actigraphy; VAS hunger ratings	∼ RMR; ∼ EE; ∼ physical activity ∼ hunger; ↑ food intake (12%)*^a^*; especially ↑ saturated fat intake in females (62%)
Tasali *et al.*^123^ Randomized cross-over study Controlled Sedentary activities	4 females, 5 males (*n* = 9) 20–31 64.2 kg; 19–24 kg/m^2^ NA 1 week regular bedtimes^*d*^	1: 2 nights of undisturbed sleep^*e*^ 2: 3 nights of disturbed sleep by acoustic stimuli during SWS (∼33 micro-arousals/h)^*e*^ Conditions ≥ 4 weeks apart	ivGTT; heart rate variability 24 h, every 20 min	↑ insulin resistance (25%) ∼ cortisol; ∼ daytime; ∼ nocturnal ↑ sympathovagal balance (14%)
Thomas *et al.*^91^ Consecutive phases study NA NA	Men (*n* = 17) 24.7 ± 2.8 NA ≥ 7 days of 7-h 45 min bedtimes^*d*^	1: 3 nights of 7-h 45-min bedtimes^*e*^ 2: 85 h of TSD (only results from 24 h shown)	[18] FDG and PET, every 24 h	↓ global cerebral glucose metabolic rate (8%), especially the thalamus, prefrontal and posterior parietal cortices
Van Cauter *et al.*^45^ Consecutive phases study Only breakfast on study day Bed rest	Men (*n* = 8) 22 – 27 22.7 ± 0.7 kg/m^2^ 1 week 23:00–07:00 bedtimes^*c*^	1: 2 nights of 8-h bedtimes^*e*^ 2: 28 h of TSD	Continuous glucose infusion during phase 2 24 h, every 30 min	↓ nocturnal GH pulses (70%)*^a^*∼ cortisol, ↓ acrophase (4%);^1^ 1 hour earlier nadir
VanHelder *et al.*^89^ Consecutive phases study Isocaloric controlled Sedentary and exercise	Men (*n* = 10) 22 ± 3 74.5 ± 11.8 kg 1 night of 7-h bedtimes^*e*^	60 h of TSD followed by 7-h of recovery sleep^*e*^ 1: sedentary activities 2: daily exercise Conditions ≥ 10 days apart	OGTT at 10 h, 60 h, and after recovery sleep	↑ insulin response (21% in group 1)
Van Leeuwen *et al.*^112^ Consecutive phases study Isocaloric controlled Free outpatient movement	Men (*n* = 13) 23.1 ± 2.5 NA 2 weeks regular bedtimes^*c*^	1: 2 nights of 8-h bedtimes^*e*^ 2: 5 nights of 4-h bedtimes^*e*^	Flow cytometry, real time PCR (1 morning sample), salivary cortisol (10 times a day)	∼ cortisol ↑ IL-6 (63%) ↑ IL-1β (37%) ∼ TNF-α↑ hsCRP (45%)
Vgontas *et al.*^106^ Consecutive phases study Usual diet Free outpatient movement (sedentary during sampling)	13 females, 12 males (*n* = 25) 25.2 ± 3.8 23.8 ± 2.3 kg/m^2^ 2 weeks regular bedtimes^*d*^	1: 4 nights of 8-h bedtimes^*e*^ 2: 8 nights of 6-h bedtimes^*e*^	24 h, every 30 min (ELISA, TNF-α, and IL-6)	∼ cortisol; ↓ and 2 h earlier peak ↑ IL-6 ↑ TNF-α in males; ∼ in females

Note: Sleep curtailment was verified by ^*c*^questionnaires/diaries, ^*d*^by actigraphy, or ^*e*^by polysomnography. The percentage of change in metabolic parameters was given in the article or calculated from *^a^*absolute levels in the text/tables or from *^b^*graph values of the original article. Metabolic measurements are in plasma/serum, unless otherwise specified. Bed rest was always required during the sleep-deprived state. ∼ similar; TSD: total sleep deprivation; VAS: visual analogue scale; RMR: resting metabolic rate; IL-6: interleukin 6; IL-1β: interleukin 1β; TNF-α: tumor necrosis factor-α; EE: energy expenditure; ivGTT: intravenous glucose tolerance test; RQ: respiratory quotient; OGTT: oral glucose tolerance test; REM: rapid-eye movement; GH: growth hormone; ACTH: adrenocorticotropic hormone; FDG: fludeoxyglucose; PET: position emission tomography.

### Effects on glucose metabolism

In the course of the night, glucose supply to the brain must remain adequate in the setting of slightly declining plasma glucose levels. Infusion of [^3^H3]-labeled glucose in healthy, lean subjects revealed decreased glucose usage during the night, whereas in subjects kept awake glucose usage was increased (Table 1).^44^ Total and partial sleep deprivation reduced glucose tolerance in lean individuals. This has been documented by intravenous glucose tolerance tests (ivGTT),^101^,^109^,^114^,^123^,^124^ oral glucose tolerance tests (OGTT),^87^,^89^,^109^ insulin suppression tests with octreotide,^90^ as well as hypoglycemic and euglycemic hypoinsulinemic clamps^97^,^114^ (Table 1). Impaired glucose metabolism was not only shown in healthy study participants, but also in individuals with type 1 diabetes: after a four-hour night in adults with type 1 diabetes, glucose infusion rate decreased by 21%, as assessed by hyperinsulinemic euglycemic clamp^115^ (Table 1). In addition to altered peripheral glucose metabolism during total sleep deprivation, cerebral glucose uptake decreased, especially in the prefrontal and posterior parietal cortex and in the thalamus of healthy volunteers^91^ (Table 1). Interestingly, most studies found no compensatory rise in plasma insulin secondary to increased insulin resistance after short sleep,^109^,^114^ fragmented sleep,^123^ or total sleep deprivation^90^ (Table 1). However, increased insulin secretion in response to ivGTTs was found after 60 hours of sleep deprivation^89^ and two nights of sleep fragmentation.^124^ Thus, almost all studies find decreased glucose tolerance and a dampened compensatory insulin response after sleep curtailment.

### Effects on EE

Two groups found that one night of total sleep deprivation increased EE during the night, whereas the following day, resting and postprandial EE were reduced by 5% and 20%, respectively, overall leading to a positive energy balance^43^,^100^**(**Fig. 3 and Table 1**)**. Similarly, Schmid *et al.* reported that volunteers had decreased spontaneous physical activity after a night of short sleep compared to a night with restful sleep^110^ (Table 1). Fragmenting sleep for two consecutive nights lead to an increase in the respiratory quotient (RQ), indicating a shift from fat toward carbohydrate oxidation, without affecting total EE^125^ (Table 1). High RQ predicts weight gain and insulin resistance.^126^ Two weeks of partial sleep deprivation (5.5 hours) decreased resting metabolic rate and increased RQ in a randomized, cross-over study^109^ (Table 1). In contrast, subjects with chronic insomnia had increased metabolic rate.^127^ We have recently shown that poor sleep quality was associated with higher REE, a higher RQ, and an activation of the stress system in obese subjects with short sleep duration.^128^ In summary, EE may be differentially affected by chronic versus acute sleep curtailment and may also depend on differences in reasons of sleep curtailment (experimentally induced short sleep vs. chronic insomnia).

### Additional endocrine mechanisms linking sleep, glucose metabolism, and energy expenditure

As mentioned above, thyroid hormones, GH, cortisol, adipokines (e.g., adiponectin), and sympathovagal balance modulate glucose metabolism and energy homeostasis (Fig. 3). Acute total sleep deprivation increased TSH and free T_4_ levels,^88^ whereas partial sleep deprivation of 5.5-h nights for two weeks or six 4-h nights decreased TSH and T_4_ levels, suggesting a dose-related effect^104^,^116^ (Table 1). Decreased peripheral thyroid hormone levels are likely secondary to suppression of the nocturnal TSH peak, possibly secondary to decreased drive from TRH neurons in the PVN.^116^ Sleep-deprived rodents show reduced levels of hypothalamic TRH mRNA in the PVN of the hypothalamus.^129^ Of note, a different stressor such as acute immobilization also decreases TRH mRNA hypothalamic levels and plasma TSH levels in rodents.^130^

Insulin resistance can result from increases in insulin counter-regulatory hormones, including GH and cortisol (Fig. 3). One night of total sleep deprivation markedly decreased the GH peak that usually occurs around sleep onset^45^,^95^ (Table 1). Partial sleep deprivation did not affect 24-h levels of GH in lean or obese individuals,^101^,^109^,^118^ but six 4-h nights changed GH nocturnal secretory patterns: sleep deprivation was associated with a biphasic pattern compared to the usual single large GH peak at sleep onset.^102^ This extended exposure to GH may decrease glucose uptake in muscles, thus contributing to insulin resistance. However, the threshold of GH levels contributing to insulin resistance is unknown. In contrast to the previous study, decreased GH levels during the first four hours of sleep were reported after a two-week partial sleep-deprivation period, in association with more SWS.^109^,^118^ GH is usually secreted during SWS, thus this unexpected finding may be due to a dissociation of GH secretion from SWS. In summary, the effects of sleep deprivation on GH plasma levels are variable.

Partial and total sleep deprivation resulted in elevated salivary and plasma cortisol the following afternoon and evening^100^,^101^,^109^,^114^,^117^ (Table 1), while morning cortisol levels may be unchanged or even decreased.^90^,^97^,^117^ As awakening is usually associated with a cortisol surge, this finding probably reflects the lack of the awakening response. In addition, a delay and/or a reduction in the acrophase of cortisol have been reported after partial and total sleep deprivation.^95^,^102^,^109^ In a sleep-deprived state, cortisol levels decreased more slowly after reaching their acrophase, possibly indicating decreased sensitivity to the negative feedback effects on the hypothalamic–pituitary–adrenal axis. Mean 24-h levels of plasma cortisol often remain unchanged by sleep deprivation,^43^,^95^,^96^,^99^,^106^,^109^,^123^ but the circadian cortisol-pattern changes, as described above.

Adiponectin levels were similar after partial and total sleep deprivation and sleep fragmentation^99^,^121^,^124^ (Table 1). However, subgroup analysis revealed that effects of sleep restriction on adiponectin levels may be gender and race dependent: adiponectin decreased in Caucasian women and increased in African American women, while adiponectin levels in males did not change after short sleep^121^ (Table 1). Besides influencing energy homeostasis, decreased adiponectin levels worsen insulin resistance.

Sympathovagal balance, defined as the ratio between the activity of the sympathetic and the parasympathic nervous system, can be indirectly assessed with heart rate variability analysis. Since the activity of this system is very diverse, on the basis of the different anatomic branches, it is unclear to which extent perturbations in heart-rate variability may be predictive of changes in basal metabolic rate and insulin sensitivity. Furthermore, heart rate, EPI and NE levels are markers of sympathetic activity. Acute sleep deprivation is associated with increased sympathetic activity, decreased parasympathetic tone and therefore, with increased sympathovagal balance^101^,^109^,^123^,^124^**(**Table 1**)**. Total EE is usually decreased in acute sleep deprivation; thus, other mechanisms, such as decreased thyroid hormones or adiponectin levels, must prevail over increased sympathovagal tone **(**Fig. 3**)**. Increased sympathovagal balance also adds to insulin resistance^131^ (Fig. 3). Furthermore, activation of the sympathetic nervous system and inhibition of the parasympathetic nerves decreases insulin secretion; thus, changes in the autonomic nervous system could explain the absence of a compensatory insulin response after sleep curtailment.^132^

In summary, sleep loss impairs glucose metabolism and may decrease EE. This is mediated by multiple factors, such as decreased thyroid hormones and, possibly, adiponectin levels; increased sympathovagal balance; and altered patterns of cortisol secretion, while the role of GH is still unclear (Fig. 3).

### Effects on appetite

It is not clear to what extent obesity in subjects with short sleep is caused by increased energy intake versus decreased EE^133^ (Fig. 3 and Table 1). If food is provided *ad libitum*, partial or total sleep deprivation may not change hunger ratings, while likely increasing food intake.^99^,^117^,^120^ On the other hand, when food intake is controlled, sleep deprivation usually increases appetite. Self-reported sleep quality was inversely related to appetite in 53 first-degree, normal weight relatives of subjects with diabetes.^134^ After two 4-h nights, there was a 24% increase in daytime appetite ratings;^105^ in addition, sleep deprivation specifically increased appetite for sweet and salty foods. The effects of sleep deprivation on appetite may not be immediate: one 4-h night of sleep deprivation was associated with increases in food intake 36 h later, while this parameter was unaffected immediately following sleep deprivation.^113^ In addition, the amount of sleep deprivation needed to increase hunger ratings may be variable. One night of total, but not partial, sleep deprivation, significantly increased hunger ratings in normal weight males.^98^,^113^ No differences in total food intake were observed after two 4-h, 15 min- versus 8-h, 15 min- nights, but subjects ate relatively more fat when sleeping less.^119^ In a randomized, cross-over, five-day study of 4 h versus 9 h of sleep, with a controlled diet on days 1–4 versus an *ad libitum* diet on day five, women, but not men, exhibited increased total caloric intake and a preference for fatty foods, especially if saturated, suggesting that women may be more susceptible to the orexic effects of sleep deprivation than men.^122^

Chronotype, the individual attribute determining morning or evening preference, modulates metabolism. Obese subjects carrying the CLOCK 3111TC/CC polymorphism, a variant of one of the core clock genes associated with being an “owl” or an evening person, sleep 20 minutes less.^135^ In addition, these subjects had a higher intake of trans-fatty acids and proteins, and consumed relatively later in the day, while having the same total energy intake of noncarriers. Subjects carrying the CLOCK 3111TC/CC genotype are also more resistant to weight loss: they lost about 2 kg less while on a hypocaloric diet for 30 weeks.^135^

Leptin and ghrelin inhibit and stimulate appetite, respectively (Fig. 3). Following partial sleep deprivation, mean daytime values of leptin were approximately 18% lower compared to the rested state.^104^,^105^ In addition, the circadian amplitude of leptin was dampened and the acrophase was decreased and phase advanced by two hours. A decreased circadian amplitude was also observed after three nights of total sleep deprivation.^94^ In a large cohort study of 1,024 subjects, shorter habitual sleep duration as assessed by sleep diaries related to lower morning values of leptin.^5^ Some studies found no association between short sleep and leptin levels.^94^,^98^,^100^,^119^,^136^ On the contrary, morning leptin levels were found to be increased after five nights of four hours,^120^ after one night of three hours^117^ and after one night of total sleep deprivation.^99^ Short sleep duration, as assessed by one night of polysomnography in 561 subjects, also related to higher morning leptin levels.^137^ It is difficult to explain the inconsistent results of these different studies. Of note, studies that found decreased leptin after short sleep controlled food intake, whereas when sleep-deprived subjects were allowed to eat *ad libitum*, leptin levels were unchanged or even increased.

In obese subjects, leptin levels are elevated but several of its actions are impaired, a concept referred to as *leptin resistance*. In a cross-over study design, no association among habitual sleep duration, measured actigraphically, and morning leptin levels was observed in obese subjects.^138^ Mean 24-h leptin levels in obese subjects on a controlled diet were also unchanged after 14 nights of 5.5 h versus 8.5 hours.^118^

The physiological increase in total ghrelin levels in the early night was blunted when individuals were kept awake.^95^ A similar phenomenon is reported in chronic insomniacs.^135^ However, morning total ghrelin levels were increased after one night of sleep deprivation^95^,^98^ and in individuals with shorter habitual sleep durations.^5^ Mean daytime total ghrelin levels were higher after two nights of 4-h sleep.^105^ CLOCK 3111TC/CC carriers, who display an evening preference and sleep 20 min shorter, also had increased morning ghrelin levels.^134^ Mean 24-h levels of the metabolically active acetylated ghrelin were higher in obese subjects after two weeks of 5.5-h nights.^118^ Overall, sleep curtailment is consistently associated with higher daytime ghrelin.

The effect of sleep deprivation on the orexin system in normal subjects is not well studied. However, it is known that patients with narcolepsy have abnormally low orexin-A CSF levels, which correlate both with body weight and sleep abnormality.^139^ Total sleep deprivation increased orexin levels in the CSF of squirrel monkeys, and increased cFos expression in orexin neurons in rats.^81^,^140^ It is challenging to assess the activity of the orexin system in humans. Central determination requires collection of CSF, an invasive technique. Determination of orexin in plasma is hampered by the lack of adequate assays, in terms of sensitivity and specificity. Hyperactivity of the orexin system could contribute to increased food intake during sleep deprivation. In summary, appetite and food intake are often increased following sleep curtailment, possibly via decreased leptin, increased ghrelin and a hyperactive orexin system (Fig. 3).

### Effects on the immune system

Enhanced levels of circulating leucocytes, pro-inflammatory cytokines (e.g., IL-6, TNF-α, IL-1β) and CRP have been associated with an increased risk of cardiovascular disease and type 2 diabetes in long-term human and experimental studies.^141^–^144^ Increased plasma levels of IL-6 were reported after partial and total sleep deprivation in most cases,^93^,^106^,^107^,^108^,^112^ although two studies only reported a loss of circadian rhythms^82^,^96^ (Table 1). IL-1β was elevated after partial,^106^,^112^ and total sleep deprivation,^96^ but one study did not find any changes after total sleep deprivation^82^ (Table 1). TNF-α levels are most often,^82^,^93^,^112^ but not always,^107^ unchanged after partial sleep restriction. One study only found increases in men, but not in women, after partial sleep deprivation^106^ (Table 1). Another study reported higher levels of soluble TNF-α receptor I, through which TNF-α exerts its sleep promoting function.^93^ Effects of sleep curtailment on CRP levels are inconsistent: they have been reported lower,^96^ unchanged,^124^ or higher.^108^,^112^ Prolonged total sleep deprivation has been associated with leucocytosis^82^ (Table 1). Thus, differences in study outcomes may be influenced by individual or gender differences. In addition, sensitivity and specificity of commercial cytokine assays are highly variable. Highly sensitive analytical methods, such as mass spectrometry, may be needed for accurate cytokine measurements.^145^ We conclude that short sleep promotes a proinflammatory state, which, in turn, exerts its negative consequences on insulin resistance.

## The other side of the coin: metabolic dysfunction influencing sleep

Most of the studies of sleep deprivation have been performed in healthy, lean subjects and therefore could not address the effect of sleep deprivation on subjects with obesity and diabetes. Obesity and diabetes are often associated with increased stress or differences in life style that could negatively influence sleep^146^ (Fig. 3). Depression and obesity may occur together as well: obesity may negatively affect mood and both pharmacologic antidepressant therapy and major depression itself are associated with weight gain.^147^ We recently reported that obese subjects often suffer from sleep-disordered breathing, which induces frequent microawakenings and loss of SWS.^148^ Similar effects of BMI on sleep have also been observed in youth. Decreased total sleep time and less sleep efficiency were observed in severely obese adolescents, of which 74% displayed sleep apnea (16.5 years; BMI 60.3 ± 2.1 kg/m^2^).^149^ Changes in sleep architecture in obesity have been reported: severely obese adolescents (12–15 years; BMI 50.9; 39.8–63.0 kg/m^2^, apnea index 14 episodes/h) have a decrease in REM sleep.^150^

Symptoms associated with diabetes, such as thirst, nocturia, extreme glucose excursions, and mood alterations, may also interfere with sleep, independent of obesity. To this point, adults with type 1 diabetes, who are typically not obese, report a poorer sleep quality than their matched controls without diabetes.^151^ These awakenings were associated with a rapid decline of glucose levels, but not with the absolute values of hypoglycemia.^152^ Furthermore, studies in animals suggest that diet composition may directly influence sleep: mice fed a high-fat diet display an attenuated rhythm in the circadian expression of core clock genes.^40^ Of note, augmented amplitude of the peripheral and central circadian rhythms favors wakefulness during the day and sleep at night, whereas an attenuated circadian amplitude disrupts the sleep–wake cycle.^153^ Likewise, studies in rodents demonstrate that physical activity at the congruent time (i.e., nocturnal for mice) increases the central circadian amplitude, suggesting that active behavior during the day and sleep at night in humans may be beneficial for high-amplitude circadian rhythms.^154^ Thus, sleep and circadian rhythm are bidirectionally connected (Fig. 3). Leptin influences sleep architecture in normal fed rodents, increasing SWS by 13% and decreasing REM by 30%.^155^ Furthermore, *ob/ob* mice that lack leptin and *db/db* mice that lack the leptin receptor, both display sleep disturbances that are reverted by leptin replacement. In summary, metabolic dysfunction, altered hormone levels, and sleep abnormalities probably all influence each other.^156^,^157^

In summary, sleep deprivation and obesity may potentiate each other in a vicious reverberating circuit; short sleep may induce weight gain, via the mechanisms outlined in this paper, whereas obesity, in turn, via sleep apnea and other symptoms, may disrupt sleep. Observational studies, especially if cross-sectional, may not be well suited to disentangle these aggregate, causative factors. This underlies the need for behavioral, life-style intervention studies.

## Possible therapeutic role of melatonin

In nonequatorial areas, the duration, intensity, and spectral quality of natural light vary across seasons.^158^ For example, during a summer in England, there was relatively more blue light versus green and red light exposure, in addition to increased light intensity and duration.^158^ These are important signals for seasonal rhythms, transmitted to the body via the duration of nocturnal melatonin secretion. As a consequence of artificial lighting and other environmental modifications, seasonality is greatly attenuated in modern humans. Furthermore, because sleep is usually the only time during which humans do not experience light exposure, shorter sleep duration will result in longer light exposure. Light suppresses the pineal production of melatonin in a dose-related fashion. White artificial light greater than 200 lux completely suppressed nocturnal plasma melatonin production, dim room light (106 lux) reduced melatonin by 88% and light intensities lower than 80 lux did not affect melatonin levels.^159^ As the night progresses, the intensity of continuous artificial light needed to suppress circulating melatonin increases.^160^ Of note, light of shorter wavelength, such as the light that is typically emitted by electronic devices such as televisions and computers, induces relatively more melatonin suppression and likely leads to greater sleep disruption.^160^

Urinary 24-h melatonin concentrations decreased by more than 50% when subjects slept five hours versus eight hours, while being continuously exposed to white light (700 lux) during wake hours.^161^ Diminished levels of melatonin were likely due to three additional hours of light exposure. Likewise, mean 24-h plasma melatonin concentrations were decreased after 6 nights of 4-h versus 12-h bedtimes.^103^ The onset of melatonin secretion was delayed, due to four hours longer light exposure before bedtime (300 lux) and the acrophase was reduced in sleep-deprived subjects.

Recent studies suggest that melatonin may have a physiological role in modulating metabolism. For example, removal of the pineal gland decreased the responsiveness of several insulin-dependent hepatic kinases during the dark phase in rats.^162^ This effect was reversed by nocturnal supplementation of melatonin in the drinking water. Chronic melatonin supplementation in drinking water also reduced body weight in rodents.^163^,^164^ Patients with metabolic syndrome have disturbances in melatonin production, characterized by an alteration of the night melatonin-insulin ratio of unclear clinical significance.^165^ In a large genome-wide association studies conducted in individuals of European origin, certain genetic variants of the melatonin receptor 1B were associated with an increased risk of type 2 diabetes.^166^–^168^ However, the most common receptor variant did not influence the relationship between sleep abnormalities and type 2 diabetes.^166^ Interestingly, the melatonin receptor 1B is present on pancreatic β-cells, and melatonin has an inhibitory effect on insulin secretion. Thus, it was feared that treatment with melatonin in subjects with diabetes would increase blood glucose levels. On the other hand, since type 2 diabetes is associated with reduced melatonin levels toward the end of the night;^169^ thus, there may be a physiological rationale for melatonin supplementation at bedtime. Few studies have addressed these questions. Sleep efficiency improved after five months of treatment with 2 mg prolonged-release melatonin administered two hours before bedtime, while total sleep time was unaffected in 26 subjects with type 2 diabetes.^170^ HbA1c levels also improved (8.47% vs. 9.13% at baseline). Lack of a control group prevented understanding whether improved HbA1c levels were in part due to other factors than melatonin administration, such as better pharmacological and diet compliance due to study participation. In lean subjects, melatonin administration at night for 2 months was associated with decreased LDL levels and improved oxidative status.^171^ Another study found a positive correlation between plasma melatonin levels at 2 am and HDL levels in 36 women treated with 1 mg melatonin for one month.^172^ As melatonin promotes sleep, the decrease in obesity and improvement in insulin resistance could be due to longer and better sleep per se, but it could also relate to other melatonin-dependent pathways yet to be identified (Fig. 3).

## Discussion and future directions

Epidemiological and laboratory data prove the existence of a relationship between short or disturbed sleep and adverse metabolic outcomes. In general, these effects are small in size, but certain specific populations may be more susceptible than others. For example, age plays a clear role as the relationship between short sleep and obesity is stronger in children. Sleep duration and sleep architecture change with age in healthy individuals, with SWS becoming less common with age.^173^ It is also well known that sex and reproductive hormones affects sleep regulatory mechanisms^174^ and that sleep disorder affects women differently from men.^175^ It is unfortunate that experimental studies, in both humans and in rodents, are mostly conducted in the male gender, thus unduly discriminating the female gender.

Similarly, sleep deprivation is more common in minorities but the effects of socioeconomic status per se versus ethnicity remain to be determined. Individual susceptibility within these categories varies greatly: identification of contributing factors will greatly help prevention and treatment and be one of the tenets of individualized medicine.^176^ Few negative effects are expected to be observed in individuals with voluntary sleep curtailment (natural short sleepers), in contrast to individuals with short sleep due to social or work-related pressure or chronic insomnia. Current epidemiologic studies often lack an objective measurement of sleep duration, such as actigraphy or repeated sleep diaries. Thus, prospective studies with objective sleep duration measurements are warranted in different subgroups to further explore this relationship.

In addition, the causality of the relationship between sleep and metabolic disorders remains unclear, even in prospective studies. Obese/diabetic individuals may be sleeping less than lean subjects because of differences in life style or stress; their sleep could be interrupted more often by symptoms of disturbed glucose levels or sleep apnea; evidence arises that possibly circulating hormones affect the brain inducing worsened sleep (Fig. 3). As already mentioned, causality can be addressed best in long-term studies where “prescribed” sleep duration is randomized, but this may present with practical difficulties and significant challenges, including the fact that the biological need for optimal sleep duration varies and cannot be estimated at an individual level. Sleep is regulated by circadian and homeostatic mechanisms. As far as homeostatic mechanisms are concerned, it has been the matter of a long scientific debate whether during wakefulness, single or rather multiple metabolites accumulate, progressively increasing the pressure to sleep during the day. Classic experiments of parabiosis seem to support the notion of endogenous “sleep-promoting” substances. Recently, the importance of adenosinergic neurotransmission in the regulation of sleep, especially non-REM sleep, has been underlined.^177^ Nevertheless, it is certain that there is a large individual variability in sleep needs. Sensitive and specific biomarkers of sleep deprivation are needed for various reasons, including clinical and medico-legal reasons.^178^

The effect of sleep extension in subsets of short sleepers on metabolism can be addressed in randomized controlled trials. Currently, we are conducting such a study in short-sleeping obese individuals.^179^ Investigating the effects of sleep extension in other subgroups of varying age, BMI, and ethnicity will also be of interest. The effect of metabolic disturbances on sleep, such as hyperinsulinemia or increased circulating levels of proinflammatory cytokines, may be empirically addressed in polysomnographical studies. As it is difficult to artificially mimic the internal milieu of obese/diabetic individuals, effects of metabolism may be modeled in animal studies using parabiosis, connecting circulations of lean and obese animals and comparing sleep characteristics.

As indicated by studies of sleep deprivation, the mechanisms connecting short sleep and obesity/insulin resistance are probably mediated by three pathways: increases in appetite, decreases in EE, and influences on glucose metabolism (Fig. 3). Endocrine mechanisms influencing these pathways are complex and interconnected. The existing studies of sleep deprivation have generated variable results; small sample size, differences in the control of food-intake and physical activity levels, and inherent individual differences may have all contributed to this variability. In addition, these studies only addressed the effects of acute sleep deprivation and were not performed in real-life situations. We suggest that future studies should focus on the effects of sleep deprivation in specific patient populations, including obese/insulin-resistant individuals and chronic insomniacs versus natural short sleepers, and should mimic real-life conditions.

Most studies find worsened glucose tolerance after sleep curtailment, which may be beneficial from a teleological perspective, at least in the short term. When our ancestors remained awake during the night, they were probably experiencing a threat (e.g., absence of food, presence of predators), so inducing a state in which glucose availability was advantageous. Likewise, increases in appetite result in more food intake. Together with reduced EE, this will further increase blood glucose availability. In modern societies, however, prolonged exposure to such a state may result in obesity and type 2 diabetes.

At this point, there is a general consensus that short sleep has negative effects on health, but the amount of sleep curtailment causing metabolic disturbances is unclear, and specific groups at greater risk are yet to be identified. Whether melatonin would benefit obese/diabetic subjects, or a subset of these individuals, remains to be proven in rigorous randomized clinical trials. Supplementation of melatonin has been shown to improve sleep efficiency, and HbA1c levels in patients with type 2 diabetes.^170^ Lean subjects may also profit from melatonin administration as well, as it improved lipid profiles.^171^,^172^ Thus, melatonin appears to be a promising supplement in battling the obesity/diabetes epidemics, but controlled studies need to confirm its positive effect on metabolism. Furthermore, the long-term effects and side effects of chronic melatonin supplementation need to be examined in different subgroups.

Overall, improving sleep duration and quality is a potential tool to counteract the epidemics of obesity and diabetes. However, before sleep extension advice is translated to the clinic, more research is needed.
